# Decaying Spruce Wood as a Factor in Soil Carbon and Energy Flow Through Microbial Communities

**DOI:** 10.1111/1758-2229.70236

**Published:** 2025-11-27

**Authors:** Adam Górski, Ewa Błońska, Rafał Ważny, Jarosław Lasota

**Affiliations:** ^1^ Department of Ecology and Silviculture, Faculty of Forestry University of Agriculture in Krakow Kraków Poland; ^2^ Małopolska Centre of Biotechnology Jagiellonian University in Kraków Kraków Poland

**Keywords:** forest soils, microorganisms structure, mountain ecosystems, spruce, woody debris

## Abstract

Climate change poses significant challenges to forest ecosystems, particularly influencing processes such as deadwood decomposition and carbon sequestration. This study explores the impact of decaying spruce wood on soil properties, enzymatic activity and microbial structure across an altitudinal gradient in mountain ecosystems dominated by spruce monocultures. In the Babia Góra Massif (Poland), we analysed soils beneath highly decomposed spruce logs (600–1200 m a.s.l.), focusing on soil chemistry, enzymatic activity and microbial composition. Decaying wood enriches soil with carbon and nitrogen, boosting β‐glucosidase and phosphatase activities. Increased soil moisture content under decaying wood promotes decomposition and microbial activity. Interestingly, microbial community composition under deadwood exhibited biodiversity changes compared to control soils, and metabolic activity was notably higher, suggesting shifts in microbial function rather than community diversity. The study highlights the significant role of decaying spruce wood in shaping soil properties and microbial processes in mountain ecosystems, emphasising its contribution to carbon and nitrogen enrichment and enhanced enzymatic activities. These findings underscore the ecological importance of deadwood in forest ecosystems, particularly in the context of carbon cycling and climate change adaptation. Sustainable forest management practices should prioritise the retention of deadwood to maintain vital ecosystem functions, particularly in the context of global climate change. Future studies should broaden this approach by including different tree species and additional environmental factors, in order to better understand the variability and resilience of deadwood‐driven soil processes across forest ecosystems.

## Graphical abstract

Decomposing spruce deadwood enhances microbial activity, increases carbon and nitrogen availability and improves soil moisture retention. It plays a crucial role in long‐term carbon storage and biodiversity maintenance, influencing energy and nutrient cycling within forest ecosystems.

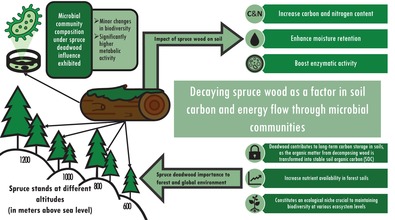

## 1 Introduction

Deadwood is an integral part of forest ecosystems, contributing significantly to the biogeochemical processes occurring therein (Müller and Bütler 2010; Dufour‐Pelletier et al. 2020). With the ongoing climate change and increasing anthropogenic pressure on the natural environment, the role of deadwood in carbon sequestration and maintaining ecosystem functions is becoming increasingly important (Błońska et al. 2023). Deadwood is of great importance, especially in the context of the role it plays in the carbon cycle and energy flow in ecosystems (Stokland et al. 2012; Stienen et al. 2014; Piaszczyk et al. 2022). Deadwood actively affects the soil microenvironment. These microenvironments become habitats for many rare and specialised species of organisms that cannot survive in other conditions (Lassauce et al. 2011). The decomposition processes of dead biomass are driven by diverse groups of microorganisms (Tedersoo et al. 2003; Gómez‐Brandón et al. 2020). Microorganisms, which are the main decomposers in ecosystems, decompose organic matter, transforming it into forms available to other organisms and contributing to long‐term carbon sequestration in the soil (Błońska et al. 2024). Some of this carbon is mineralised, leading to carbon dioxide emissions into the atmosphere, but a significant part is stored as stable organic compounds in the soil (Błońska et al. 2022). This process is crucial for the long‐term maintenance of carbon resources in forest ecosystems and is important in the context of global climate change (Russell et al. 2015; Lasota et al. 2022). Deadwood plays a critical role in shaping soil biodiversity by providing food resources, habitat and specific microenvironments; its slow decomposition supports distinct microbial communities and environmental conditions that are absent in other parts of the ecosystem (Harmon et al. 1986; Herrmann et al. 2015; Bani et al. 2018; Bardelli et al. 2018; Błońska et al. 2024; Zumr et al. 2024). Deadwood traits (tree species, age and position) strongly shape chemical properties, microbial biomass, moisture and enzymatic activity via fungal communities, ultimately determining decomposition rates in temperate forest ecosystems (Jomura et al. 2022). According to Pioli et al. (2023) bacterial alpha diversity was influenced by both decay stage and log characteristics, whereas bacterial beta diversity was primarily determined by log diameter.

One of the most important challenges facing the modern world is climate change, and research on the dynamics of deadwood decomposition and its ability to sequester carbon is essential to understand how to increase the potential of forests to absorb carbon. Changes in temperature and humidity, as well as more frequent extreme weather events, can affect the rate of deadwood decomposition and, consequently, carbon sequestration processes. Research on the impact of decaying wood on soil properties will provide essential knowledge that can help develop sustainable strategies for managing forest resources, including estimating the appropriate amount of deadwood in forests. Our research concerns mountain ecosystems with spruce monocultures, which lose stability and often collapse under the conditions of changing climate. This research is an extension of the experiment concerning beech wood, the results of which were published earlier (Błońska et al. 2024). The extension of this research is even more important because of the profound differences between the species. This manifests in the fact that they are phylogenetically different, that they contain different substances that inhibit decomposition, that they have different groups of pathogens and destructors, and that they have different chemical properties. Spruce was selected for this study because its wood differs chemically from broadleaved species such as beech, containing higher amounts of lignin and resin. These compounds can slow decomposition and inhibit microbial colonisation and enzymatic activity, making spruce a particularly relevant model species for assessing the impact of deadwood on soil microbial communities. Previous research has shown that, for these various reasons, dead spruce wood decomposes at a slower rate than beech wood (Bujoczek 2012). The aim of our research was to determine the impact of highly decomposed spruce wood on basic soil properties, enzymatic activity and microbial structure at different locations along the height gradient. The following research hypotheses were tested: (1) decaying spruce wood affects basic physicochemical properties of soils, such as pH, C and N content, regardless of the position in the altitude gradient; (2) enzymatic activity reflects the effect of released substrates from decaying spruce wood, regardless of the position in the altitude gradient and (3) the structure of soil microorganisms differs significantly between soils under the influence of decaying spruce wood and soils without such wood, and these differences are dependent on the position in the altitude gradient. Our research follows the work of previous studies on deadwood decomposition (Błońska et al. 2023, 2024), which analysed both advanced stages of spruce and beech decomposition. Using a similar research design allows for direct comparison of the obtained results. As a result, our research significantly contributes to a better understanding of the differences in the rates and mechanisms of decomposition of wood of different tree species in mountain ecosystems.

## 2 Experimental Procedures

### 2.1 Study Sites and Experiment Design

The research was conducted on the southern and northern slopes of the Babia Góra Massif (49°35′18″ N; 19°32′23″E). Three transects were selected along an altitudinal gradient for the study. In each transect, research plots were located at elevations of 600, 800, 1000 and 1200 m a.s.l. The transects, with sampling points, were spaced 500 m apart. In total, the study covered 72 research plots (2 expositions × 4 elevations × 3 transects × 3 replicates = 72 study plots). The transects were situated in a complex of Dystric Cambisols with a silty loam texture. The dominant vegetation on the plots consisted of mixed fir, spruce and beech stands with similar canopy density (Błońska et al. 2024). The research sites were in mature forest areas that had not been used for agriculture in the past. When selecting study plots, areas affected by erosion, landslides and colluvial processes were intentionally avoided. The average annual temperature for the study plots at 600 m a.s.l. was 6.0°C, at 800 m a.s.l. it was 5.1°C, at 1000 m a.s.l. it was 4.0°C and at 1200 m a.s.l. it was 3.1°C. Sampling was carried out according to the scheme presented in the article by Błońska et al. (2024). On each study plot in the transects, one spruce log was selected with a diameter of at least 25–35 cm at the centre. The examined logs were highly decomposed, reaching the fifth stage of decomposition, as determined using a standardised five‐point decay scale. Previous research suggests that wood in stage V decomposition has the most significant impact on soil properties (Herrmann et al. 2015; Piaszczyk, Błońska, and Lasota 2019; Piaszczyk, Błońska, Lasota, et al. 2019). The research covered wood in the fifth stage of decomposition, as at this stage the decomposition processes are most advanced and their impact on the soil microenvironment and the functioning of microorganisms is most visible, making it possible to assess the maximum ecological effect of the presence of dead wood. The degree of decomposition was determined using the classification by Maser et al. (1979), which was also applied in previous studies (Lasota et al. 2018). Wood samples (D) were taken from the central part of the log using a metal sampler (box) with dimensions of 7 × 7 × 7 cm. Soil samples (UD) were collected directly beneath the log using a small shovel. Additionally, a soil sample was taken 1 m away from the log, which served as a control (C). Control soil samples were collected 1 m away from the log, as our previous studies have shown that this distance is sufficient to exclude the influence of deadwood (Piaszczyk, Błońska, and Lasota 2019; Piaszczyk, Błońska, Lasota, et al. 2019). Soil samples were collected from a depth of 0–10 cm beneath the log, and control soil samples were taken from the same depth range of 0–10 cm. To collect a soil sample beneath the log, a fragment of the wood was removed and the soil surface was carefully cleaned of wood debris down to the humus horizon. One sample was taken from each transect, from each height, each exposition and from each type of sample for determination of fungi and bacteria (1 sample × 3 transects × 4 elevations × 3 types of sample × 2 expositions = 72). Three samples were taken from each transect from each height, each exposition and from each type of sample for determining basic properties (3 samples × 3 transects × 3 elevations × 3 types of sample × 2 expositions = 162). Field and laboratory tests were conducted in 2021.

### 2.2 Basic Laboratory Analysis

The fresh samples were first weighed in their natural state. They were then dried at 105°C for 48 h and reweighed. Instantaneous moisture content was calculated as the difference between fresh and dry mass, expressed relative to the dry mass. The pH in H_2_O for both wood and soil samples was measured using the potentiometric technique. The levels of carbon (C) and nitrogen (N) were analysed using a LECO CNS device (TrueMac Analyser Leco, St. Joseph, MI, USA). The C/N ratio was then calculated from these C and N concentrations. Lignin content in the wood and soil samples was assessed through a spectrophotometric method, following the procedure described by Rodrigues et al. (1999) and Antczak et al. (2013).

### 2.3 Enzymes Activity

Soil samples with their natural moisture content were collected to assess enzymatic activity. Upon arrival at the laboratory, the samples were stored at 4°C. The enzymatic activity was measured using the fluorescence method. The activity of extracellular enzymes—β‐glucosidase (BG), N‐acetyl‐β‐d‐glucosaminidase (NAG), phosphatase (PH) was analysed according to the protocols described by Pritsch et al. (2004), Turner (2010) and Sannaullah et al. (Sanaullah et al. 2016). The activity of β‐glucosidase (BG), N‐acetyl‐β‐d‐glucosaminidase (NAG) and phosphatase (PH) was analysed as these enzymes are key indicators of microbial activity, reflecting cellulose degradation (BG), nitrogen mineralisation (NAG) and phosphorus release (PH) during organic matter decomposition.

### 2.4 Fungal and Bacterial DNA Library Preparation and Processing of NGS Data

DNA was isolated from the following sample types: deadwood (D), soil under deadwood (UD) and control soil (C). Control soil samples were collected 1 m from decaying logs at four altitudes (600, 800, 1000 and 1200 m above sea level) on both North and South exposures. DNA was extracted from 5 g of sample (*n* = 3, total 72 samples). Wood samples were homogenised with a mortar in the liquid nitrogen. The sample was incubated in 30 mL of CTAB and 64 μL of β‐mercaptoethanol at 64°C for 60 min at 300 rpm. Subsequently, 215 μL of RNase was added, and after incubation at room temperature (RT) for 10 min, the sample was centrifuged at 7000*g* for 5 min. Then, 20 mL of the supernatant was mixed with 20 mL of chloroform and centrifuged at 7000*g* for 30 min. The supernatant was transferred to a new tube, 20 mL of isopropanol was added and the sample was incubated at −80°C overnight. The sample was then centrifuged at 7000*g* at 4°C for 40 min. The pelleted nucleic acid was washed twice with 70% ethanol, dried at RT and suspended in 200 μL of TE buffer (Tris‐EDTA). DNA was purified using the MagMAX Microbiome Ultra Nucleic Acid Isolation Kit (Thermo Fisher Scientific). Fungal and bacterial libraries were prepared following the methods described in Małek et al. (2021) and Ważny et al. (2023). The ITS1 rDNA region was amplified for fungal community profiling, while the V5–V7 region of 16S rDNA was amplified for bacterial community profiling. DNA libraries were sequenced on the Illumina NovaSeq platform (2 × 250 bp paired end) by Novogene (UK). Sequencing depth was 100,000 reads per sample. Paired end reads were merged using FLASH (Magoč and Salzberg 2011). Chimera sequences were detected and removed with UCHIME algorithm (Edgar et al. 2011). Then effective tags were denoised with DADA2 module using QIIME2 software (Caporaso et al. 2010) to obtain initial amplicon sequence variants (ASVs). In the next step, ASVs were rarefied in QIIME2 to standardise the number of sequencing reads. The rarefaction depth was 31,532 for fungi and 30,702 for bacteria. Species annotation was performed with UNITE database (version no. 9.0) for fungi and Silva database (version no. 138) for bacteria. ASVs were filtered for very low abundance and frequency. ASVs with a relative abundance of at least 0.01% and a frequency of at least three samples were used in further analysis. This application resulted in a 49% and 53% decrease of ASVs for fungi and bacteria, respectively.

### 2.5 Statistical Analysis

Spearman's correlation coefficient was used to determine the relationship between the enzymatic activity and basic chemical properties which may change because of the impact of decaying wood. Principal component analysis (PCA) was used to confirm the relationship between the tested properties and to group the analysed samples with respect to their type and altitude. The grouping was based on enzymatic activity and basic chemical properties. PCA analysis was used to confirm the distinctiveness of soils that are characterised by improved soil health through the impact of deadwood. The Shapiro–Wilk test was used to assess normality, and Levene's test was used to check the homogeneity of variances. The Kruskal–Wallis and post hoc test was used to assess the differences between the analysed sample types and altitude. Generalised linear mixed‐effects model (GLMM) was used to determine the role of sample type and altitude and the interaction between them on the number of fungal and bacterial taxa. Statistical tests were used to indicate the significance of differences in parameters indicating improved soil health (enzymatic activity, C and N concentration, pH). Statistical analyses were performed using the programming language R (R Core Team 2016) in R Studio (RStudio Team 2020). The readxl, dplyr and ggplot2 packages were used to prepare graphs with chemical properties and enzymatic activity. The corrplot package was used to create a correlation plot, while the MASS, factoextra and ggfortify packages were used to perform PCA.

Alpha diversity was estimated based on the number of ASVs per sample that passed an abundance threshold. GLMM was used to test the significant differences in ASV richness among study sites. Bray–Curtis and Manhattan distances were calculated to assess community composition differences, among groups (exposition, altitude and sample type). Principal coordinate analysis (PCoA) based on Bray–Curtis dissimilarities, calculated from log 2‐transformed relative ASV abundances, was used to visualise sample differences in multi‐dimensional scale. ANOSIM and PERMANOVA tests were used to assess the statistical significance of differences between study sites, as visualised in the PCoA plot. A heatmap, generated using the ‘superheat’ R package, was used to visualise relative ASV abundances (scaled) across samples. Dendrograms, based on Manhattan distances, were included to cluster samples. FUNGuild database2 was used to analyse the functional groups of fungi identified in the study (Nguyen et al. 2016).

## 3 Results

Soil pH values ranged from 3.46 to 4.94, with variations depending on altitude, sample type and exposure (Table 1). In the northern exposure, the lowest pH was observed in control soils at 600 m a.s.l. (3.46), while the highest pH was recorded in control soils at 1000 m a.s.l. (4.27). In the southern exposure, pH was generally higher, with the highest value observed in control soils at 800 m a.s.l. (4.94). Overall, the influence of altitude on pH was significant (*F* = 9.07, *p* = 0.0036). Nitrogen content varied significantly among sample types, with the highest concentration found in under‐wood (UD) samples at 600 m a.s.l. in northern exposure (1.94%), while the lowest concentration was detected directly in wood (D) samples at 800 m a.s.l. in northern exposure (0.26%). Sample type had a significant effect on nitrogen concentration (*F* = 30.59, *p* = 0.0001). The carbon content was highest directly in wood (D) samples across all altitudes. For instance, at 800 m a.s.l. in northern exposure, directly in wood samples had a carbon content of 59.99%, compared to only 8.91% in control soils. Sample type significantly affected carbon content (*F* = 208.99, *p* = 0.0001). Interestingly, altitude had no significant influence on carbon content (*F* = 1.29, *p* = 0.2596). Soil moisture content ranged from 35.73% to 82.50%, with wood samples showing the highest moisture levels. The impact of sample type on moisture was significant (*F* = 71.01, *p* = 0.0001). Exposure, sample type and altitude significantly influenced lignin content (Table 1).

**TABLE 1:** Basic properties of different sample types in different localisation and GLM analysis results (mean ± SD; *n* = 3; N—nitrogen content (%); C—carbon content (%); moisture (%); lignin content (mg cm^−3^)).

Exposure	Altitude	Sample type	pH	N	C	Moisture	Lignin
N	600	C	3.46 ± 0.03	0.85 ± 0.11	15.21 ± 2.40	51.31 ± 7.65	195.13 ± 10.94
		D	3.82 ± 0.03	0.54 ± 0.10	52.50 ± 4.35	74.91 ± 2.66	72.94 ± 8.59
		UD	3.50 ± 0.07	1.94 ± 0.16	47.16 ± 6.26	72.87 ± 6.67	47.21 ± 21.41
	800	C	3.82 ± 0.06	0.51 ± 0.14	8.91 ± 3.11	43.34 ± 2.41	204.65 ± 7.10
		D	3.95 ± 0.04	0.26 ± 0.06	59.99 ± 0.66	82.50 ± 1.41	101.14 ± 7.44
		UD	3.83 ± 0.07	0.78 ± 0.35	31.95 ± 7.66	59.09 ± 7.31	157.97 ± 32.19
	1000	C	4.27 ± 0.04	0.48 ± 0.02	6.20 ± 0.47	35.73 ± 0.21	87.81 ± 23.54
		D	3.93 ± 0.04	0.40 ± 0.01	57.95 ± 0.27	77.64 ± 0.28	94.92 ± 9.70
		UD	4.15 ± 0.06	0.48 ± 0.09	15.76 ± 3.91	46.12 ± 3.22	68.06 ± 11.80
	1200	C	3.50 ± 0.02	0.58 ± 0.03	10.99 ± 0.63	43.49 ± 4.38	99.26 ± 22.54
		D	3.74 ± 0.06	0.27 ± 0.02	57.15 ± 1.00	82.95 ± 1.25	80.75 ± 8.41
		UD	3.48 ± 0.04	1.87 ± 0.04	54.25 ± 1.44	72.57 ± 0.77	33.06 ± 4.54
S	600	C	4.40 ± 0.10	0.66 ± 0.10	12.57 ± 2.13	63.04 ± 5.08	127.70 ± 21.70
		D	4.07 ± 0.19	0.57 ± 0.34	53.20 ± 1.08	80.07 ± 0.91	67.01 ± 7.54
		UD	4.06 ± 0.13	1.56 ± 0.13	39.79 ± 4.16	48.21 ± 9.72	44.43 ± 6.74
	800	C	4.94 ± 0.02	0.52 ± 0.03	9.17 ± 0.95	37.97 ± 1.97	118.87 ± 6.11
		D	4.29 ± 0.25	0.56 ± 0.14	55.05 ± 1.06	73.09 ± 0.42	72.84 ± 6.77
		UD	3.90 ± 0.05	1.19 ± 0.09	32.74 ± 2.29	57.28 ± 0.04	52.86 ± 11.02
	1000	C	3.40 ± 0.02	0.45 ± 0.11	10.28 ± 2.37	38.36 ± 5.52	76.66 ± 33.68
		D	3.70 ± 0.14	0.89 ± 0.09	52.52 ± 0.37	75.47 ± 0.66	54.53 ± 1.03
		UD	3.39 ± 0.03	1.04 ± 0.08	24.36 ± 2.70	54.67 ± 2.69	64.95 ± 5.49
	1200	C	3.43 ± 0.04	0.65 ± 0.09	13.65 ± 3.46	62.04 ± 6.50	94.29 ± 27.73
		D	3.86 ± 0.10	0.98 ± 0.17	50.98 ± 1.25	81.20 ± 0.52	45.92 ± 2.15
		UD	3.67 ± 0.06	0.92 ± 0.04	25.74 ± 0.53	70.08 ± 4.82	36.23 ± 4.86

	*F*	*p*	*F*	*p*	*F*	*P*	*F*	*P*	*F*	*p*
Exposure	2.72	0.1034	1.22	0.2720	3.23	0.0767	0.02	0.9681	17.41	**0.0001**
Sample type	1.77	0.1771	30.59	**0.0001**	208.99	**0.0001**	71.01	**0.0001**	24.95	**0.0001**
Altitude	9.07	**0.0036**	1.52	0.2218	1.29	0.2596	0.48	0.4905	13.31	**0.0005**
Exposure × Altitude × Sample type	0.37	0.6896	4.77	**0.0116**	2.49	0.0906	2.39	0.0996	0.17	0.8416

*Note:* Statistically significant effects (*p* < 0.05) are shown in bold.

**FIGURE 1:**
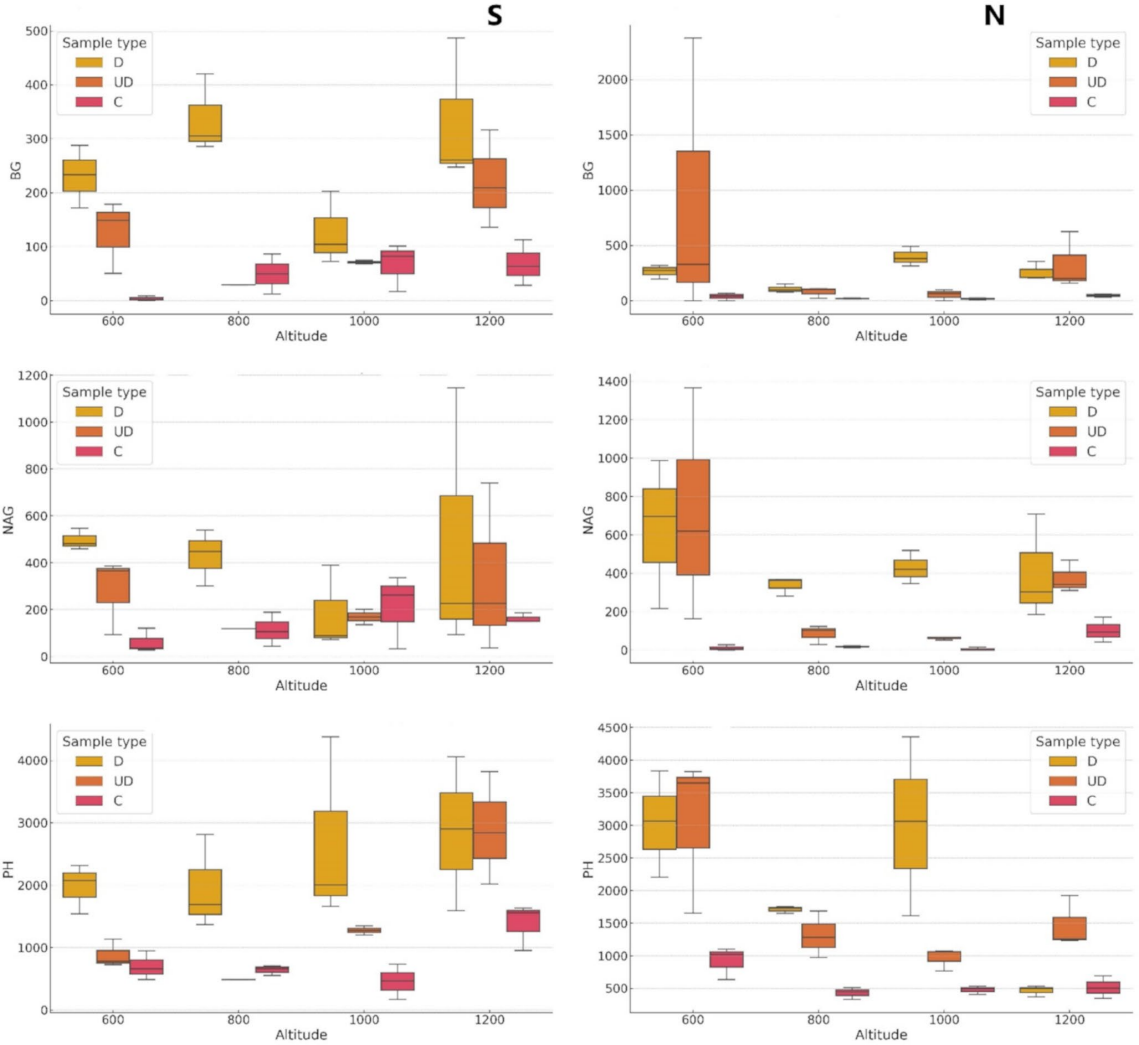
Enzymatic activity (nmol MUB g^−1^ h^−1^) depending on sample type (deadwood [D] soil [C] and soil under deadwood [UD]) and position in the altitude gradient (600, 800, 1000 and 1200 m a.s.l.) exposure (S—south, N—north) (BG—β‐glucosidase, NAG—N‐acetyl‐β‐glucosaminidase, PH—phosphatase).

Figure 1 illustrates the enzymatic activity (BG, NAG and PH) depending on sample type and altitude. It demonstrates that wood samples and soils under the influence of decaying wood exhibited higher enzymatic activities, regardless of altitude. Table 2 presents the results of the GLM analysis for enzymatic activity, focusing on three key enzymes: β‐glucosidase (BG), N‐acetyl‐β‐d‐glucosaminidase (NAG) and phosphatase (PH). The results show that sample type had a significant effect on the activity of all enzymes, while altitude and exposure had no significant effect. BG activity was significantly higher in wood samples and soils directly under decaying wood compared to control soils, with sample type showing a significant effect (*F* = 4.04, *p* = 0.0221). Sample type significantly affected NAG activity (*F* = 12.10, *p* = 0.0001). PH activity was also higher in under‐wood soils across all altitudes, with sample type having a significant effect (*F* = 16.28, *p* = 0.0001).

**TABLE 2:** GLM analysis results for enzymatic activity.

	BG		NAG		PH	
	*F*	*p*	*F*	*p*	*F*	*P*
Exposure	1.08	0.3016	0.03	0.8668	0.10	0.7508
Sample type	4.04	**0.0221**	12.10	**0.0001**	16.28	**0.0001**
Altitude	0.20	0.6551	0.49	0.4853	0.01	0.9208
Exposure × Altitude × Sample type	0.75	0.4753	0.75	0.4739	0.53	0.5508

*Note:* Statistically significant effects (*p* < 0.05) are shown in bold.

**FIGURE 2:**
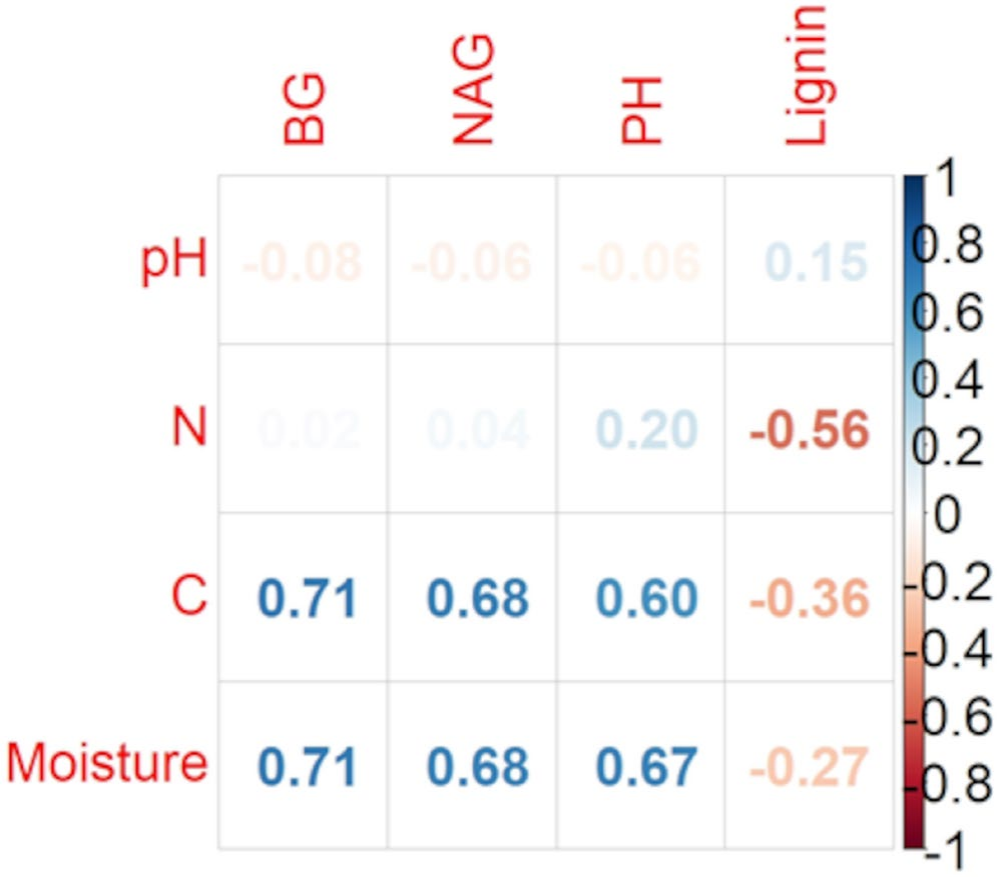
Correlations between the examined properties of wood and soil (BG—β‐glucosidase, NAG—N‐acetyl‐β‐glucosaminidase, PH—phosphatase, C—carbon concentration, N—nitrogen concentration).

The analysis revealed strong positive correlations between carbon concentration and enzymatic activity, particularly for β‐glucosidase and phosphatase. Nitrogen content was also positively correlated with enzyme activity, though to a lesser extent. The correlation between soil pH and enzymatic activity was weaker (Figure 2).

### 3.1 Fungal Diversity

**FIGURE 3:**
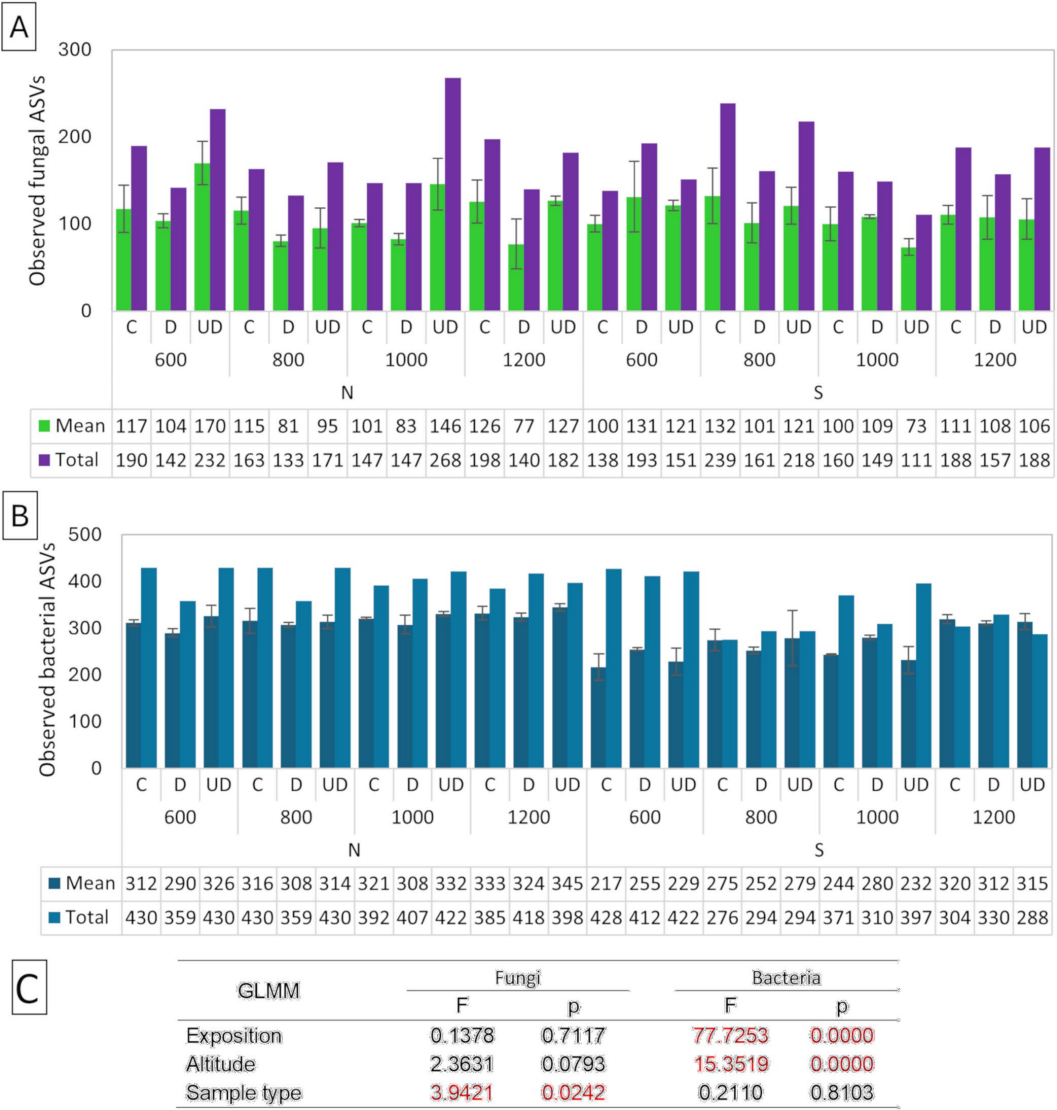
Alpha diversity of fungal (A) and bacterial (B) ASVs identified in deadwood (D) in soil (C) and in soil under deadwood (UD) at the different altitudes (600, 800, 1000 and 1200 m above sea level) and exposure (S—south, N—north). (C) Results of generalised linear mixed‐effects model (GLMM) used to determine the role of exposure, altitude and sample type on the number of fungal and bacterial taxa.

**FIGURE 4:**
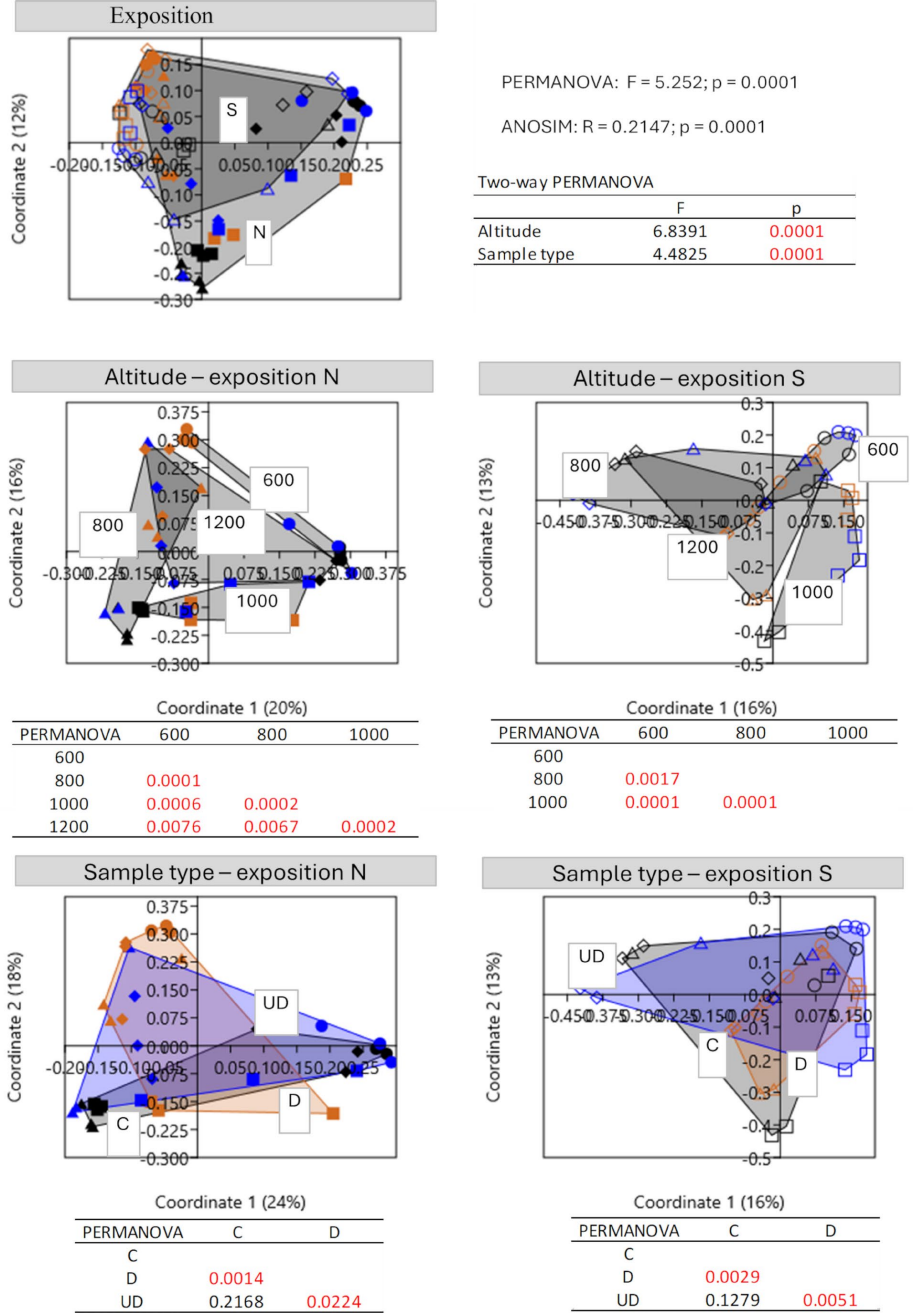
Principal coordinates analysis (PCoA) based on Bray–Curtis distances and *p* values of PERMANOVA multivariate tests between samples of deadwood (D) soil (C) and soil under deadwood (UD) collected at the different altitudes (600, 800, 1000 and 1200 m above sea level) calculated for fungal ASVs (log 2(RA + 1)).

The alpha diversity of the fungal community was estimated on 490 observed ASVs. The number of identified fungal ASVs differed significantly depending on the sample type (GLMM: *p* = 0.0242), but not on exposition (GLMM: *p* = 0.7117) and altitude (GLMM: *p* = 0.0793) (Figure 3). Focusing solely on sample type, a significantly higher number of taxa were identified in soil under deadwood (mean 120 ASVs) and soil (mean 113 ASVs) than in deadwood (mean 99 ASVs) (Figure 4). Principal coordinate analysis (PCoA) calculated on the basis of Bray–Curtis distances showed that all tested variables (exposure, altitude and sample type) affected the fungal community structures (Figure 4). The significant effect of exposure was confirmed by PERMANOVA (*F* = 5.252; *p* = 0.0001) and ANOSIM (*R* = 0.2147; *p* = 0.0001). Next, two‐way PERMANOVA analysis of ASVs revealed that the fungal communities were affected by altitude and by sample type (Figure 4). Each pairwise comparison for altitude was statistically significant. However, in both exposures, the fungal communities in soil under deadwood did not differ from the control soil (N: *p* = 0,2168; S: *p* = 0.1279). Manhattan distance‐based clustering revealed distinct clusters for deadwood samples, separate from soil and soil under deadwood (Figure S1A). The fungal communities in all investigated groups were dominated by *Agaricomycetes*, *Mortierellomycetes* and *Sordariomycetes* classes (Figures S2 and S3). The mean relative abundance of *Agaricomycetes* in control soil was 43%, in deadwood samples—64% and in soil under deadwood—37%. *Mortierellomycetes* reached 13%, 10% and 19%, respectively, and *Sordariomycetes* being the third most abundant class reached 10%, 3% and 9%, respectively (Figure S3A,B). Relative abundances of other classes, *Leotiomycetes*, *Eurotiomycetes*, *Tremellomycetes*, *Archaeorhizomycetes* and *Dothideomycetes* were significantly lower (Figure S3A,B). Of the top 30 fungal ASVs, 10 belonged to the Agaricomycetes class. These included *Russula*, *Basidiodendron*, *Piloderma*, *Craterellus*, a member of the *Clavulinaceae* family, *Resinicium*, *Hyphodontia*, *Sistrotremastum* and two unidentified taxa (Figure 6A). Mortierellomycetes was represented by five ASVs from the genus *Mortierella*, and Sordariomycetes was represented by the genera *Fusarium* and *Podospora*. FUNGuild analysis carried out on the top 30 ASVs classified four of them, *Hyphodontia*, *Resinicium Sistrotremastrum* and *Basidiodendron*, as white rot saprotrophs and one of them, *Fusarium*, as a soft rot fungus (Data S1).

### 3.2 Bacterial Diversity

**FIGURE 5:**
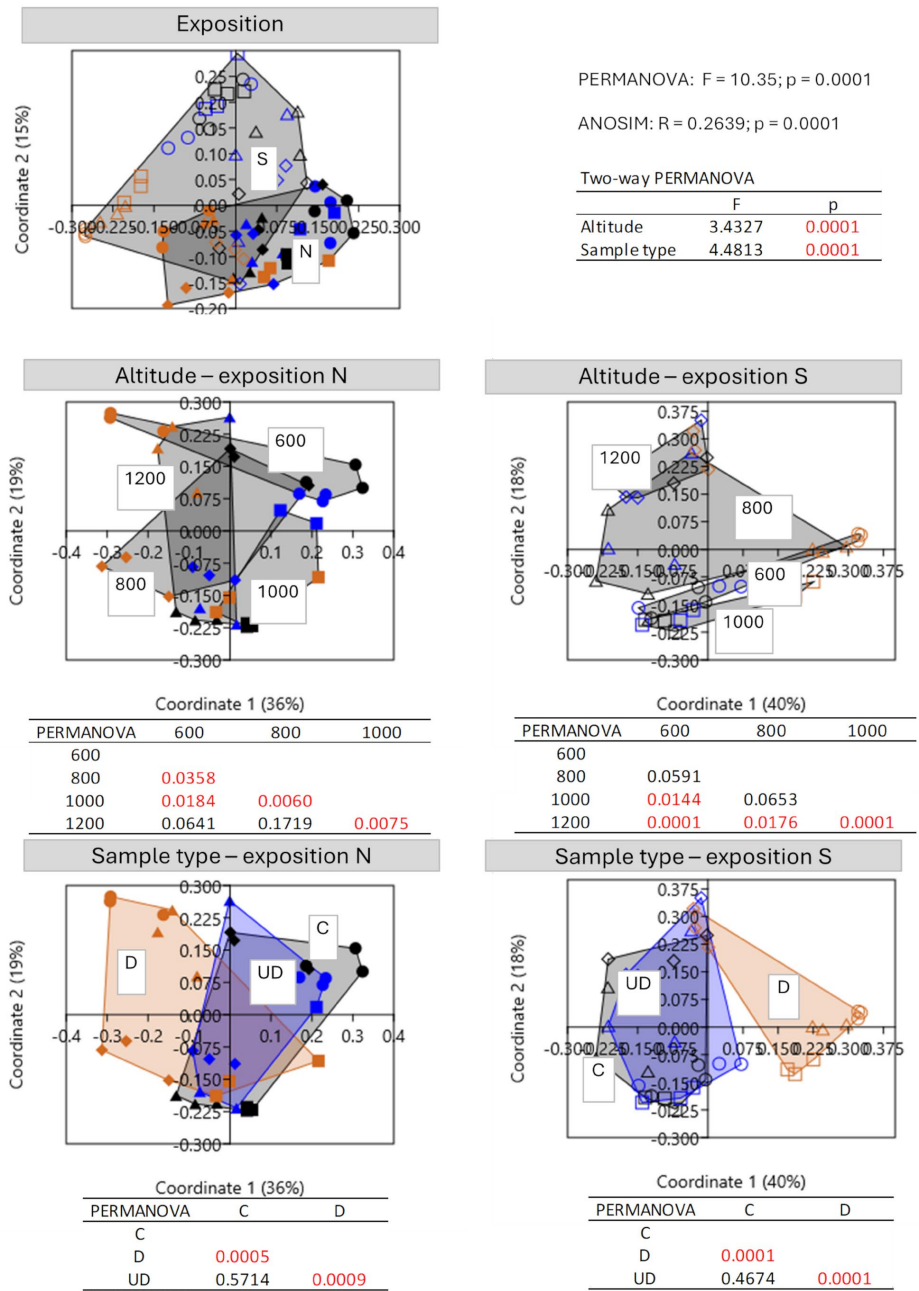
Principal coordinates analysis (PCoA) based on Bray‐Curtis distances and *p* values of PERMANOVA multivariate tests between samples of deadwood (D) soil (C) and soil under deadwood (UD) collected at the different altitudes (600, 800, 1000 and 1200 m above sea level) calculated for bacterial ASVs (log 2(RA + 1)).

**FIGURE 6:**
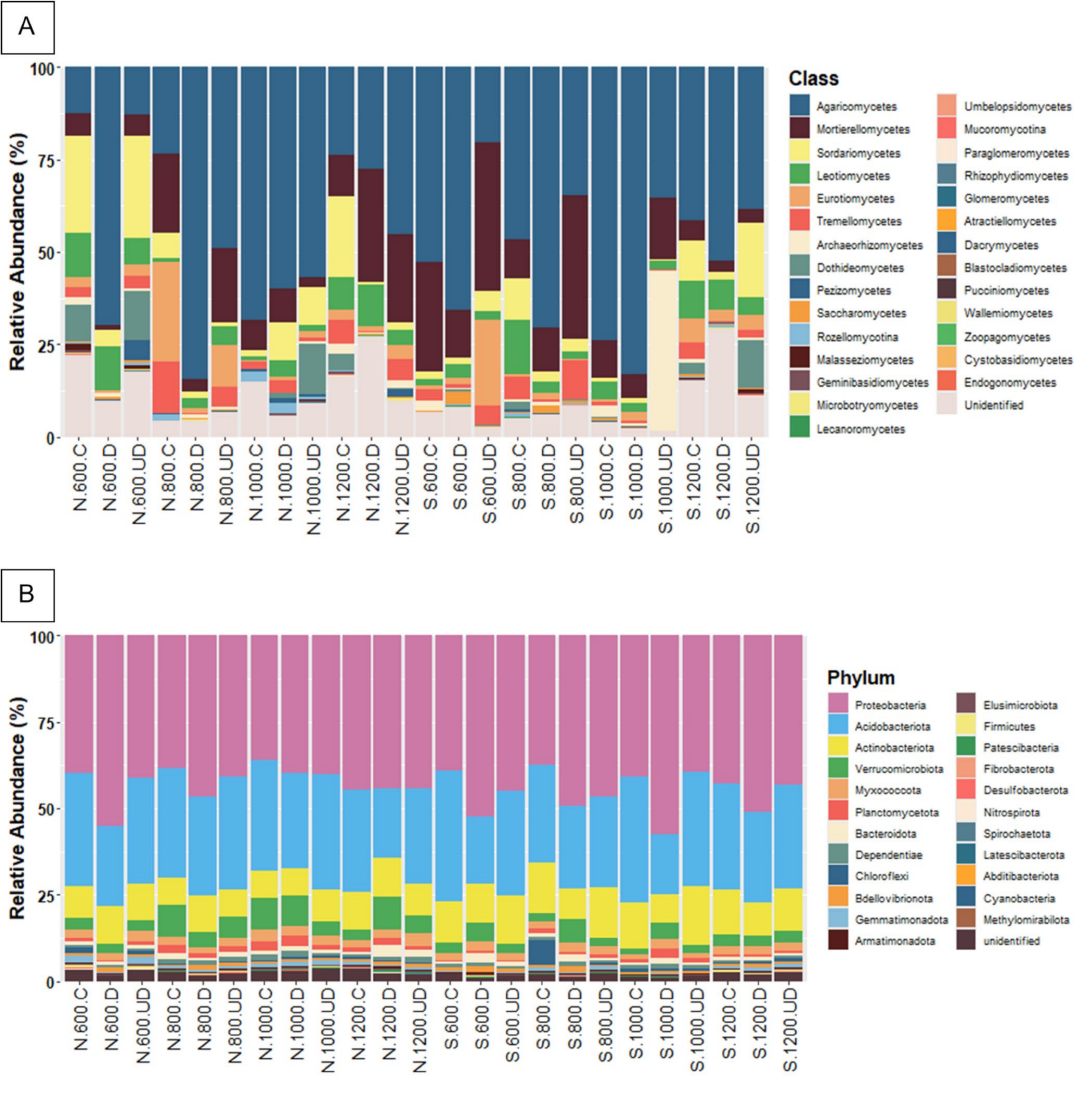
Relative abundance of fungal ASVs (A) and bacterial phyla (B) presented in deadwood (D) soil (C) and soil under deadwood (UD) collected at the different altitudes (600, 800, 1000 and 1200 m above sea level) and exposures (N—North, S—South).

A total of 638 bacteria ASVs were identified. Bacterial alpha diversity varied significantly with exposure (GLMM: *p* = 0.0000) and altitude (GLMM: *p* = 0.0000) but not sample type (GLMM: *p* = 0.8103). The mean number of bacterial ASVs varied from 217 to 345 across samples (Figure 3B,C). The North exposure (319 ASVs) had significantly higher mean ASV numbers than the South exposure (268 ASVs). Alpha diversity of bacteria was highest at 1200 m a.s.l. (324 ASVs) compared to 1000 (284 ASVs), 800 (291 ASVs) and 600 m a.s.l. (272 ASVs). Mean ASV numbers were similar across control soil, soil under deadwood and deadwood ranging from 308 to 329 in N exposure and from 263 to 274 in S exposure (Figure 3). PCoA based on Bray–Curtis distances revealed distinct bacterial community compositions among deadwood, soil under deadwood and control soil (Figure 5). PERMANOVA analyses indicated that bacterial communities of soil were determined by exposure (*p* = 0.0001), altitude (*p* = 0.0001) and sample type (*p* = 0.0001). Like fungal communities, bacterial communities in soil under deadwood were not significantly different from control soil in either exposure (N: *p* = 0.5714; S: *p* = 0.4674). The heatmap, based on Manhattan distances did not reveal distinct clustering of samples according to experimental variants (Figure S1B). The dominant bacterial phyla were Proteobacteria, Acidobacteriota and Actinobacteriota (Figure 6B; Figure S2C,D). Proteobacteria accounted for 44% in the control soil, 45% in deadwood and for 44% in soil under deadwood. Acidobacteriota reached 29%, 28% and 29%, respectively, and Actinobacteriota reached 11%, 10% and 12%, respectively.

## 4 Discussion

The results of this study clearly demonstrate the significant influence of decaying wood on soil properties and enzymatic activity in forest ecosystems, particularly along the altitudinal gradient. These findings align with previous research, which has shown that decaying wood plays a critical role in carbon cycling and nutrient availability in forest soils (Russell et al. 2015; Perreault et al. 2020; Błońska et al. 2024). By aligning our approach with that of Błońska et al. (2023, 2024), we were able to directly compare decomposition dynamics across different tree species. While previous studies focused on decomposition patterns in beech, our results emphasise the unique processes occurring in spruce‐dominated mountain ecosystems under climate‐induced instability. This comparative perspective emphasises the broader ecological implications of deadwood decomposition for forest resilience and carbon cycling. One of the key findings is the substantial increase in carbon content in soils directly under decaying wood, which was consistent across all altitudes and exposures. The significant accumulation of carbon in these soils is likely due to the organic material released during the decomposition of wood (Lajtha et al. 2018). This supports the observations of Piaszczyk et al. (2022), who found that decaying wood acts as a major source of organic matter, enriching the soil with carbon compounds that are essential for microbial processes. The increased moisture content in soils under decaying wood, as shown in our results, further supports this, as moisture retention aids in the decomposition process and enhances microbial activity (Harmon et al. 1986; Hoppe et al. 2016). The influence of decaying wood on soil pH is negligible. Soils under decaying wood exhibited a similar pH to control soils. However, it is important to note that the overall pH values remained acidic, which is characteristic of forest soils influenced by deadwood, as noted by Błońska et al. (2017). Soil pH could be the effect of the release of organic compounds from the decomposing wood.

The enhanced enzymatic activity observed in soils directly under decaying wood highlights the role of deadwood as a substrate for microbial communities. β‐glucosidase, N‐acetyl‐β‐d‐glucosaminidase and phosphatase activities were all significantly higher in these soils, indicating an increase in microbial processes related to carbon and nitrogen cycling. This finding is consistent with the work of Pritsch et al. (2004), who found that decaying wood provides a rich source of substrates for microbial enzymes, leading to elevated levels of enzymatic activity. The significant accumulation of carbon in soils under decaying wood has important implications for carbon sequestration in forest ecosystems (Shannon et al. 2022). Deadwood contributes to long‐term carbon storage in soils, as the organic matter from decomposing wood is transformed into stable soil organic carbon (SOC) fractions. This process not only sequesters carbon but also improves soil health by enhancing nutrient availability and microbial diversity. Błońska et al. (2022) similarly highlighted the role of deadwood in carbon sequestration, noting that the stable carbon fractions formed in soils under decaying wood have the potential to mitigate carbon emissions from forest ecosystems. The correlation between carbon content and enzymatic activity, as observed in this study, suggests that the presence of decaying wood stimulates microbial activity through the release of organic substrates (Małek et al. 2021). This is in line with findings by Tedersoo et al. (2003), who demonstrated that wood decomposition creates microenvironments rich in organic carbon, which in turn enhances microbial enzyme production. The correlation between enzymatic activity and moisture in soil and decaying wood is crucial for biogeochemical processes and the decomposition of organic matter (Ważny et al. 2023). Moisture strongly influences enzymatic processes, with optimal levels supporting decomposition and extremes limiting microbial activity (Błońska et al. 2024; Piaszczyk et al. 2022). Both in soil and in deadwood, optimal moisture promotes intensive decomposition of organic substances by enzymes, supporting the activity of microorganisms. Furthermore, the positive correlation between nitrogen content and enzyme activity reinforces the idea that decaying wood not only contributes to carbon cycling but also plays a role in nitrogen transformation processes, as noted by Bani et al. (2018). Interestingly, altitude had little effect on the enzymatic activity in this study. This suggests that the influence of decaying wood on microbial processes is consistent across different elevations, due to the uniform role of deadwood as a carbon and nutrient source, regardless of climatic variations associated with altitude. This finding contrasts with studies like Zumr et al. (2024), where altitude significantly influenced the decomposition rates and microbial activity. The difference could be due to the specific microclimatic conditions of our study area or the advanced stage of wood decomposition, which tends to homogenise the impact across altitudinal gradients, as suggested by Lasota et al. (2022). Although altitude significantly affected selected soil properties such as pH and lignin content, it did not translate into significant changes in enzymatic activity. In multivariate models, after accounting for moisture and carbon availability as the main predictors, the effect of altitude on β‐glucosidase, NAG and phosphatase activity was not statistically significant. This suggests that the influence of altitude acts indirectly, through modifications of moisture and carbon substrate quality, and is absorbed by these covariates rather than functioning as a direct driver of enzymatic activity.

Enriched microbial enzyme production in the soil under deadwood was not caused by increased biodiversity of soil microorganisms. The number of fungal taxa and bacterial taxa and the structure of bacterial and fungal communities in the soil under deadwood were similar to control soil. However, they differed from the deadwood itself. The previous study showed similar observations (Mäkipää et al. 2017). This suggests that soil microorganisms change their metabolome due to decaying wood. Our results suggest that shifts in metabolic activity, rather than changes in microbial diversity, underlie the patterns observed beneath deadwood; however, this conclusion is based on indirect evidence from extracellular enzyme assays. Direct functional analyses, such as metabolomic profiling, will be necessary to confirm and expand upon these findings (Jansson and Hofmockel 2018). The elevated enzymatic activity despite minimal compositional change may reflect phenotypic plasticity, where resident taxa alter transcriptional and metabolic profiles in response to deadwood inputs (Shade et al. 2012). Alternatively, it could result from differential gene expression within conserved taxa, fine‐scale strain turnover or activation of rare taxa (Carini et al. 2016; Louca et al. 2018). It is also highly probable that wood‐degrading fungi produce metabolites that may affect the enzyme activity of the soil under deadwood (Castaño et al. 2022). Deadwood decomposition is driven by complementary roles of fungi, which dominate the breakdown of recalcitrant carbon, and bacteria, which contribute to nitrogen accumulation through fixation, together shaping carbon and nutrient cycling in forest ecosystems (Tláskal et al. 2021). Decaying wood‐specific microorganisms may also not prefer to inhabit soil environments. We observed that decaying wood differentially affected bacterial and fungal communities inhabiting the soil. The number of fungal ASVs in soil under deadwood was similar to those in control soil. In the case of bacteria, deadwood did not affect the number of taxa. This parameter was determined solely by exposure. Higher bacterial diversity in North exposure compared to South exposure may be attributed to the generally higher soil moisture in North‐facing slopes. According to Bickel and Or (2020) soil aqueous‐phase content and connectivity play a pivotal role in shaping bacterial diversity by creating numerous disconnected habitats, especially at intermediate water contents. Soil moisture content regulates oxygen diffusion and nutrient transformation, both of which significantly influence the activation of dormant microbial cells and determine soil bacterial abundance and diversity (Ansari et al. 2023). Hypothesis 3 has not been fully confirmed at the taxonomic level, but the data suggest functional redundancy of microorganisms and the importance of environmental factors in shaping their activity.

Our power analysis indicated that, given our sample size (*n* = 3 per group), the statistical power was low (~0.10), limiting our ability to detect even medium‐to‐large effect sizes. Therefore, non‐significant results in our study should be interpreted with caution, as they may reflect insufficient statistical power rather than the absence of ecological effects. This highlights the importance of increasing replication in future studies to better capture the variability in soil fungal communities.

## 5 Conclusions

In summary, this study confirms the critical role of decaying wood in enhancing soil properties, enzymatic activity and microbial processes in forest ecosystems. Decaying wood significantly increases carbon and nitrogen content, enhances moisture retention and boosts enzymatic activity, regardless of altitude. These findings reinforce the importance of deadwood in carbon sequestration and nutrient cycling, aligning with previous research on the ecological benefits of decaying wood. Microbial community composition under deadwood exhibited minimal biodiversity changes compared to control soils, but metabolic activity was notably higher, suggesting shifts in microbial function rather than community diversity. Sustainable forest management practices should prioritise the retention of deadwood to maintain these vital ecosystem functions, particularly in the context of global climate change. A limitation of our study is that it focused only on spruce logs at the final stage of decomposition. Future research should expand to include multiple tree species, earlier decay stages, and a broader range of environmental variables. Such an approach will provide a more comprehensive understanding of how deadwood contributes to soil biodiversity and ecosystem functioning, and how these processes can be integrated into sustainable forest management under global climate change. Long‐term, multi‐year studies are indispensable to fully account for the effects of inter‐annual climatic variability on microbial processes and deadwood–soil interactions.

## Author Contributions

**Adam Górski:** investigation, data curation, formal analysis, writing – original draft, writing – review and editing. **Ewa Błońska:** funding acquisition, conceptualization, investigation, data curation, writing – review and editing, writing – original draft, formal analysis. **Rafał Ważny:** investigation, data curation, formal analysis, writing – original draft, writing – review and editing. **Jarosław Lasota:** funding acquisition, conceptualization, investigation, data curation, formal analysis, writing – original draft, writing – review and editing.

## Conflicts of Interest

The authors declare no conflicts of interest.

## Supporting information


**Figure S1:** Heatmap illustrating the relative abundance of the fungal (A) and bacterial ASVs (B) with dendrograms calculated based on Manhattan distances between samples; D—deadwood, C—soil, UD—soil under deadwood.

**Figure S2:** Relative abundance of fungal classes presented in deadwood (D) soil (C) and soil under deadwood (UD) collected at the different altitudes (600, 800, 1000 and 1200 m above sea level) and exposures (N—North, S—South).

**Figure S3:** Mean relative abundance of fungal classes (A—North exposure, B—South Exposure) and bacterial phyla (C—North exposure, D—South Exposure) presented in deadwood (D) soil (C) and soil under deadwood (UD) collected at the different altitudes (600, 800, 1000 and 1200 m above sea level) and exposures (N—North, S—South).

**Data S1:** Functional Guild (FUNGuild) analysis of the top 30 fungal ASVs.

## Data Availability

The data that support the findings of this study are openly available in Zenodo.org at https://doi.org/10.5281/zenodo.14943909.
