# Supplementary figures and images for: Decaying Spruce Wood as a Factor in Soil Carbon and Energy Flow Through Microbial Communities
Source: Environ Microbiol Rep. 2025 Nov 27;17(6):e70236. doi: 10.1111/1758-2229.70236 (PMC12658617; doi:10.1111/1758-2229.70236)

A

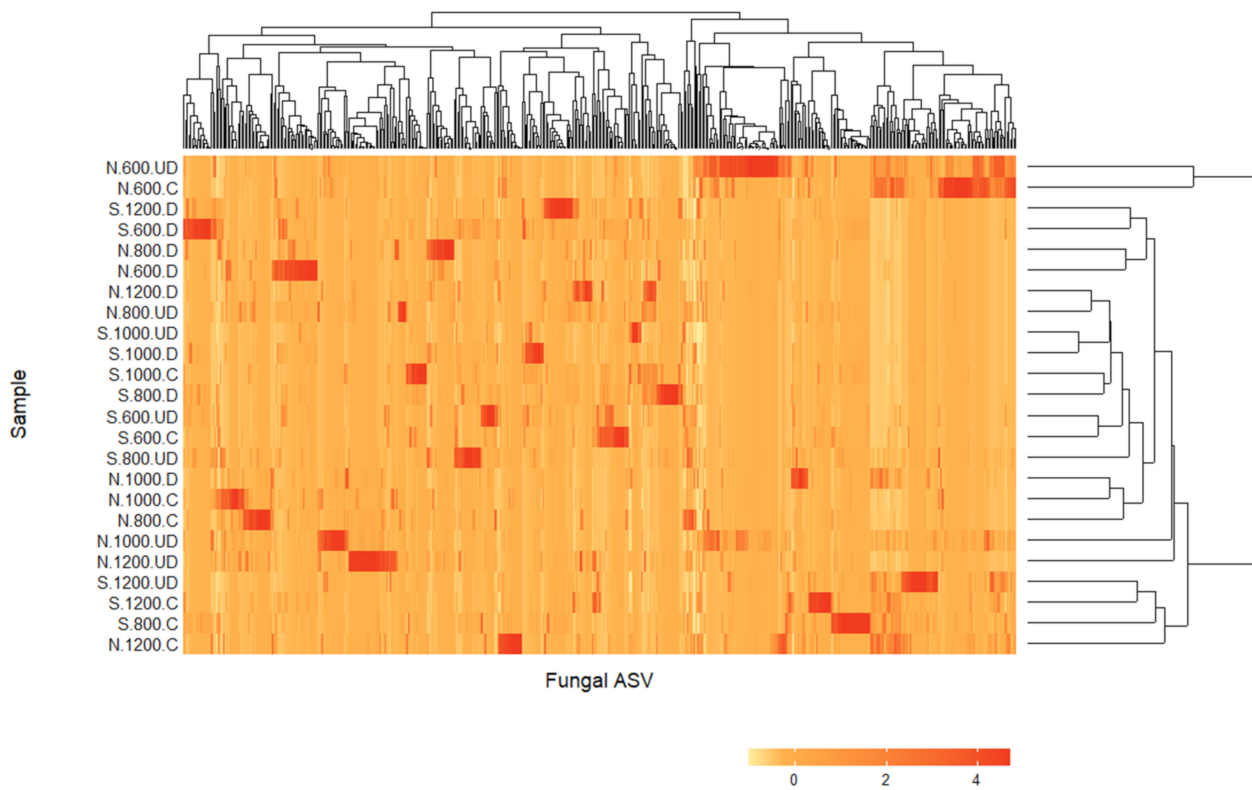

B

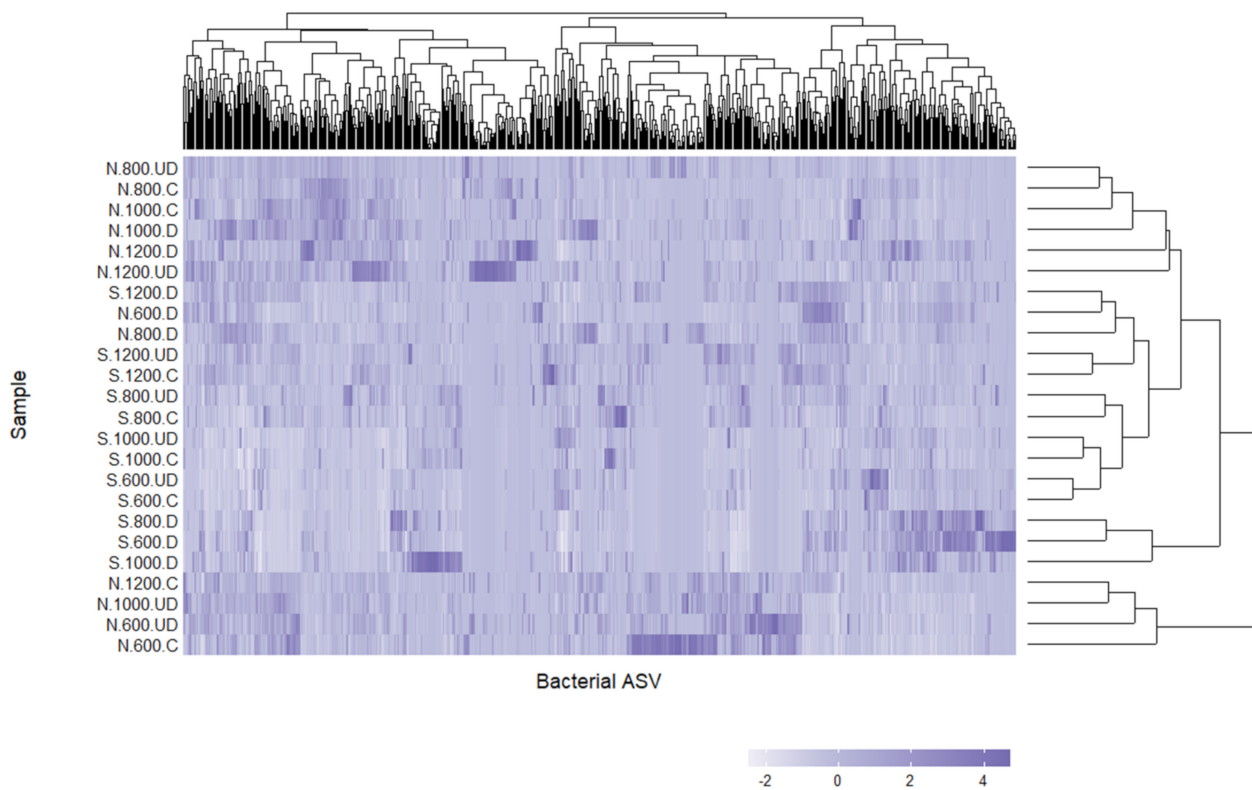

Supplement: Supplementary file 1 — Figure S1: Heatmap illustrating the relative abundance of the fungal (A) and bacterial ASVs (B) with dendrograms calculated based on Manhattan distances between samples; D—deadwood, C—soil, UD—soil under deadwood. [file EMI4-17-e70236-s002.pdf]

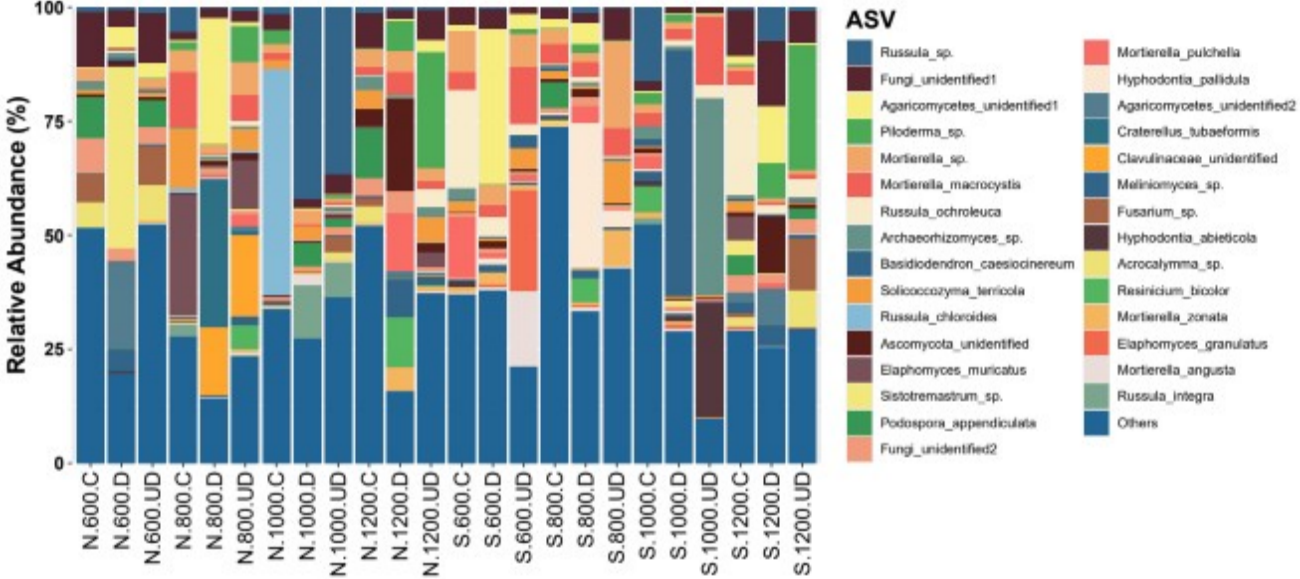

Supplement: Supplementary file 2 — Figure S2: Relative abundance of fungal classes presented in deadwood (D) soil (C) and soil under deadwood (UD) collected at the different altitudes (600, 800, 1000 and 1200 m above sea level) and exposures (N—North, S—South). [file EMI4-17-e70236-s004.pdf]

A

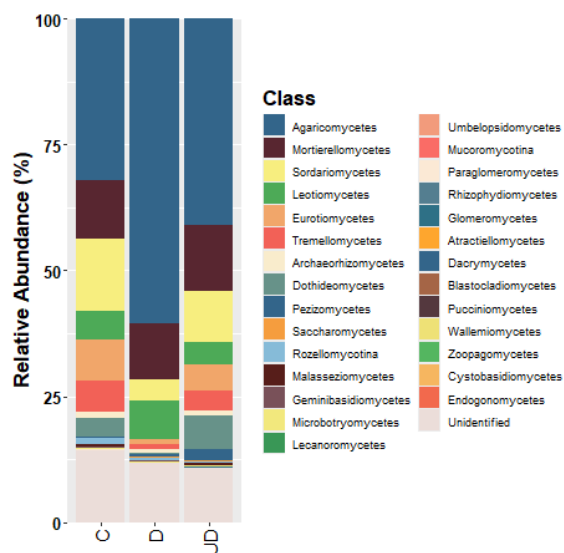

B

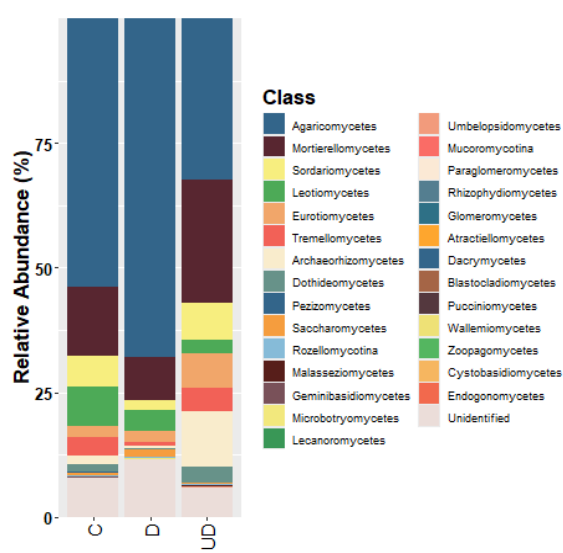

C

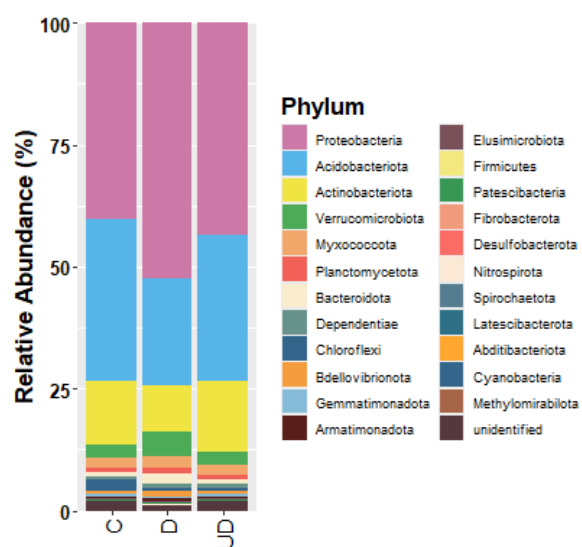

D

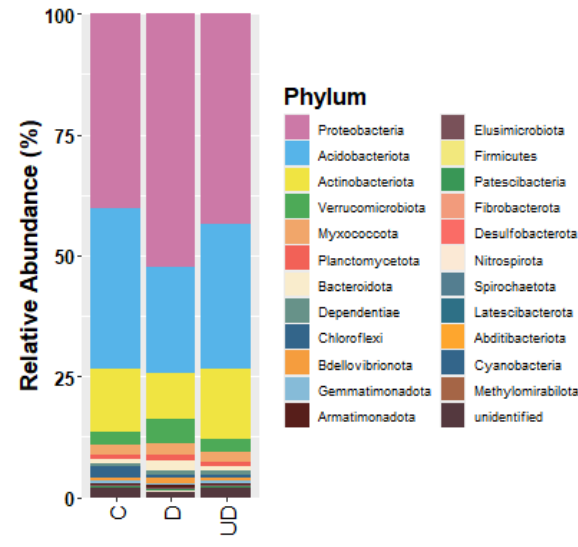

Supplement: Supplementary file 3 — Figure S3: Mean relative abundance of fungal classes (A—North exposure, B—South Exposure) and bacterial phyla (C—North exposure, D—South Exposure) presented in deadwood (D) soil (C) and soil under deadwood (UD) collected at the different altitudes (600, 800, 1000 and 1200 m above sea level) and exposures (N—North, S—South). [file EMI4-17-e70236-s001.pdf]
